# Oxidative Stress Biomarkers and Early Brain Activity in Extremely Preterm Infants: A Prospective Cohort Study

**DOI:** 10.3390/children9091376

**Published:** 2022-09-12

**Authors:** Caterina Coviello, Serafina Perrone, Giuseppe Buonocore, Simona Negro, Mariangela Longini, Floris Groenendaal, Daniel C. Vijlbrief, Carlo Dani, Manon J. N. L. Benders, Maria Luisa Tataranno

**Affiliations:** 1 Division of Neonatology, Careggi University Hospital of Florence, 50134 Florence, Italy; 2 Department of Medicine and Surgery, University of Parma, 43121 Parma, Italy; 3 Department of Molecular and Developmental Medicine, University of Siena, 53100 Siena, Italy; 4 Department of Neonatology, University Medical Center Utrecht, Utrecht University, 3508 AB Utrecht, The Netherlands

**Keywords:** F_2_-isoprostanes, amplitude-integrated EEG, preterm infants

## Abstract

Early brain activity, measured using amplitude-integrated EEG (aEEG), is correlated with neurodevelopmental outcome in preterm newborns. F_2_-isoprostanes (IPs) are early biomarkers predictive for brain damage. We aimed to investigate the relationship between perinatal IPs concentrations and quantitative aEEG measures in preterm newborns. Thirty-nine infants (gestational age (GA) 24–27 ± 6 weeks) who underwent neuromonitoring using aEEG during the first two days after birth were enrolled. The rate of spontaneous activity transients per minute (SAT rate) and inter-SAT interval (ISI) in seconds were computed. Two postnatal time-points were examined: within 12 h (day 1) and between 24 and 48 h (day 2). IPs were measured in plasma from cord blood (cb-IPs) and between 24 and 48 h (pl-IPs). Multivariable regression analyses were performed to assess the correlation between IPs and brain activity. Cb-IPs were not associated with SAT rate and ISI at day 1. Higher pl-IPs were followed by longer ISI (R = 0.68; *p* = 0.034) and decreased SAT rate (R = 0.58; *p* = 0.007) at day 2 after adjusting for GA, FiO_2_ and IVH. Higher pl-IPs levels are associated with decreased functional brain activity. Thus, pl-IPs may represent a useful biomarker of brain vulnerability in high-risk infants.

## 1. Introduction

Although advances in perinatal and neonatal care have led to increased survival rates for preterm infants, the risk of brain injury and consequent neurological and developmental impairment has remained [1]. Therefore, the prompt identification of children at high risk of developmental disabilities and the application of neuroprotective strategies to prevent brain damage are two of the main objectives of contemporary neonatology.

The preterm brain is extremely vulnerable during the early period following preterm birth. Evaluating early brain activity is a relevant biomarker of functional brain development in preterm infants [2,3]. The amplitude-integrated EEG (aEEG) is a useful bedside tool for continuous, non-invasive assessment of cerebral activity in the neonatal intensive care unit [4]. Usually, aEEG tracings are categorized on the basis of the background pattern [5]. It is well known that qualitative and quantitative aEEG parameters offer precious data about the brain function of preterm infants during early periods of their intensive care [6,7,8]. Consistently, the development of intraventricular hemorrhage (IVH) and white matter injury (WMI), the two most significant injuries that can affect the preterm brain [9], have been associated with acute changes in the aEEG during the first days of life [10,11].

The typical background pattern on conventional EEGs in preterm infants shows periods of high-voltage activity (bursts) interposed by periods of low amplitude (interburst intervals; ISIs), the so-called tracé discontinue. With increasing maturation, ISIs gradually become shorter and the bursts longer, and the trace becomes more continuous [12]. The aEEG also reflects the maturation of the developing brain, which is characterized by greater discontinuity and higher voltage in extremely preterm infants, and progressively becomes less discontinuous until achieving a continuous pattern at about 35–36 weeks of gestational age (GA) [13]. The increase in brain activity on aEEG, characterized by bursts of high voltage with rapid oscillations, interposed between periods of inactivity [14,15], known as spontaneous activity transients (SAT), is well known to be fundamental for brain development [2]. The intervals between bursts, named inter-SAT intervals (ISI) and the SATs per minute (SAT rate) [6,16] automatically calculated from the aEEG tracings, are related with brain growth and progress, and also with neurodevelopmental outcome [16].

In addition to this approach, identifying biochemical markers that come from the brain and are liberated in biological fluids may help in the early diagnosis of brain injuries and help to recognize those newborns who may benefit from prompt intervention. Despite extensive research in this field in recent years, no such biomarker has been validated in clinical practice so far. The preterm brain is particularly vulnerable to oxidative stress (OS) because rapidly growing tissues are susceptible to the harmful effects of free radicals (FRs) [17] and because the antioxidant enzyme system is still inadequate in the third trimester [18,19,20]. An important evaluation of the degree of OS is the grade of lipid peroxidation, represented by F_2_-isoprostanes (IPs), which results from free radical-induced damage by peroxidation of lipids in cell membranes. In particular, they can originate from the oxidation of eicosatetraenoic acid, a straight precursor of docosahexaenoic acid (DHA), a constituent of the cerebral cortex in the human brain, and from the oxidation of adrenic acid, highly concentrated in the myelin of the white matter of primates [21]. After an oxidative injury, prostanoids are released in the bloodstream and can be reliably measured in plasma (pl), tissues, cells, urine, cerebral spinal fluid, bile, and bronchoalveolar lavage fluid [22].

A previous study in our center demonstrated that early pl-IPs concentrations were higher in preterm newborns with WMI at term equivalent age (TEA). A cut-off level of 31.8 pg/mL could predict WMI with a sensitivity of 86% and a specificity of 60% [23]. Thus, IPs might be a significant prognostic and diagnostic tool for evaluating occurrence and severity of brain injury in newborns.

The present study aimed to assess the association between early IPs levels and early quantitative aEEG/EEG measures recorded over the first two days of life in extremely preterm infants. The demonstration of this association might help clinicians identify newborns at high risk for brain injury, start neuroprotective strategies, and monitor the progression of the disease.

## 2. Materials and Methods

### 2.1. Study Population

This was an observational, single-center study, conducted at the Neonatal Intensive Care Unit (NICU) of the Wilhelmina Children’s Hospital (Utrecht, The Netherlands). Neonates with a GA below 28 weeks, born between September 2012 and September 2014 were prospectively enrolled. Parental written informed consent was obtained, as was the approval of the local Ethics committee. This research represented a segmental study inside the protocol code 10_365 “Biomarkers and neurodevelopmental outcome”. According to local NICU clinical protocol, all infants underwent aEEG monitoring during the first two days after birth. Exclusion criteria were major congenital malformations, chromosomal disorders, inborn errors of metabolism, and morphine administration, as this is known to affect early brain activity [24].

### 2.2. Biomarkers

IPs were measured in plasma from the umbilical cord (cb-IPs) immediately after birth and between 24 and 48 h after birth (pl-IPs) with routine tests for clinical care. Plasma IPs concentrations were detected according to the LC-MS/MS method described by Casetta et al. [25].

### 2.3. Clinical Data Collection

Clinical data were collected from chart reviews of each infant, comprising GA, birth weight (BW), BW < 10th percentile (computed according to the Dutch Perinatal registry reference data [26] ), gender, mode of delivery, Apgar score at 5 min of life, fraction of inspired oxygen (FiO_2_) between 24 and 48 h after birth, occurrence and duration of mechanical ventilation, sepsis, and intraventricular hemorrhage (IVH). Sepsis was defined as patients who developed clinical signs and symptoms of an infection associated with positive blood or cerebrospinal fluid culture. IVH was graded according to the classification of Papile et al. [27]. The first cranial ultrasound was habitually executed within 6 h after birth and serially repeated until TEA.

### 2.4. AEEG Acquisition

Two-channel rawEEG and aEEG tracings were acquired using subcutaneous needle electrodes (P3-P4; F3-F4) at a sampling rate of 256 Hz. The recording was started at bedside as soon as possible after birth and continued for at least 48 h. Due to technical motives (inhomogeneity of data, impedance, differences in filters), only newborns examined with BrainZ monitors (BRM2/BRM3; Natus, Seattle, WA, USA) were enrolled. aEEG/EEG traces were visually assessed for quality by an experienced aEEG/EEG reader (MLT).

### 2.5. EEG Post-Registration Analysis

SignalBase^®^ v7.8 (University Medical Center Utrecht, The Netherlands), a locally developed software, was applied to process the raw EEG data. Two specific postnatal time-points were selected in all patients: within 12 h (day 1) and between 24 and 48 h (day 2). The aEEG records were visually assessed to identify the best hour (good quality aEEG, fewer artifacts) per time-point. Quantitative analysis with the same software program was performed to get the number of SATs per minute (SAT rate) (rounded to the nearest whole number) and the inter SAT interval (ISI, i.e., time between SAT) in seconds, both resulting from the raw EEG using a nonlinear energy operator (NLEO) (http://iopscience.iop.org/0967-3334/31/11/N02, accessed on 6 September 2022) [16]. Lastly, the percentage of time spent below 5 microvolts (% of time <5 μV) was calculated from the aEEG records.

### 2.6. Statistical Analysis

Statistical analysis was executed using IBM SPSS v 21.0 (Chicago, IL, USA). Patient characteristics were described as mean and standard deviation (SD), rate and percentage, or median and interquartile range (IQR). Univariate regression analyses were performed to evaluate the correlation between cb-IPs and pl-IPs with EEG variables. The multivariable regression model for the cb-IPs included GA, BW (percentile), and grade of IVH. The model for pl-IPs analysis included mean FiO_2_ at sampling instead of BW (percentile). IPs concentration have been proven to have an inverse correlation with GA and BW, thus these covariates were entered into the regression model [28,29]. Then, FiO_2_ was included since earlier reports demonstrated a correlation between higher oxygen administration and augmented oxidative stress biomarkers [30,31]. Next, IVH was inserted since pl-IPs concentration have been demonstrated to be associated with higher degree of IVH [32]. A *p*-value < 0.05 was statistically significant. Results were shown as coefficients of the independent variables with the 95% confidence intervals (CI).

## 3. Results

Thirty-nine infants were eligible for the study, all with good quality aEEGs. Clinical characteristics are summarized in Table 1.

None of the studied infants showed clinical or laboratory signs of perinatal asphyxia. None of the patients suffered from severe brain injury (defined as the presence of IVH grade 3 or 4), a large cerebellar hemorrhage (>3 mm), or severe white matter abnormalities on magnetic resonance imaging (MRI) (graded following the Woodward scoring system) [33]. None of the newborns received any other medication affecting brain activity, such as benzodiazepines and barbiturates.

At day 1, the mean ISI was 4 ± 1 s/min, mean SAT rate was 6 ± 1 per minute, and mean time <5 μV was 77 ± 20%. At day 2, the mean ISI was 4 ± 2 s/min, SAT rate was 6 ± 2 per minute, and mean time <5 μV was 76 ± 17%. No differences were observed between mean ISI, mean SAT rate and mean time <5 μV at day 1 and day 2 (*p* = 0.874; *p* = 0.616; *p* = 0.220, respectively).

### 3.1. Cord Blood Isoprostanes and Early Brain Activity

Univariate regression analyses showed that cb-IPs were not correlated with mean SAT rate, mean ISI, and mean time <5 μV at day 1. After adjusting for GA, BW (percentile), and grade of IVH, lower GA was associated with a longer time <5 μV at day 1 (R = 0.67; *p* = 0.015) (Table 2).

### 3.2. Isoprostanes at 24–48 h of Life and Early Brain Activity

**Figure 1:**
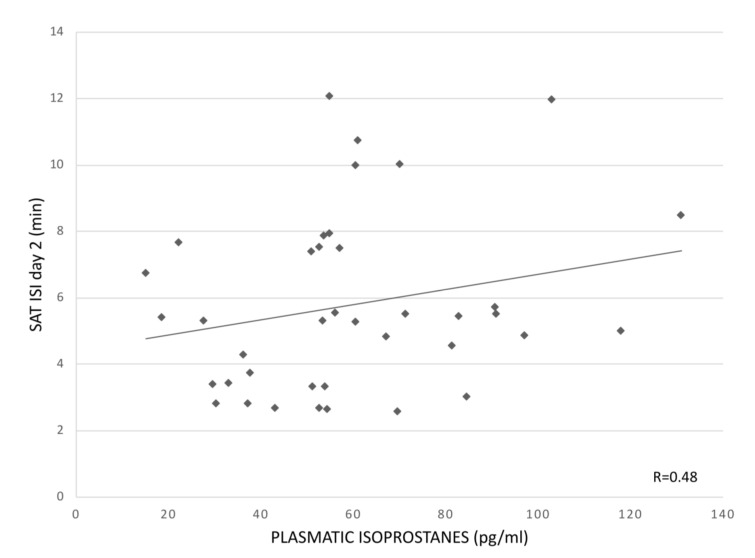
Association between pl-IPs between 24 and 48 h after birth and ISI at day 2 (sec/min) (*p* = 0.023).

**Figure 2:**
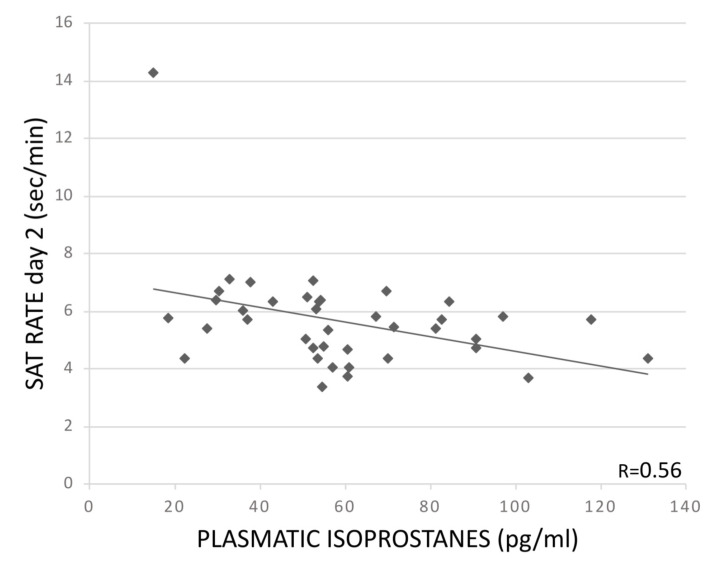
Association between pl-IPs between 24 and 48 h after birth and SAT RATE at day 2 (min) (*p* = 0.006).

The univariate regression analyses demonstrated a significant positive association between pl-IPs and ISI at day 2 (R = 0.48; *p* = 0.023). Pl-IPs were negatively correlated with SAT rate at day 2 (R = 0.56; *p* = 0.006). In the multivariable regression models, after adjusting for GA, FiO_2_ at sampling and grade of IVH, these correlations remained significant (R = 0.68; *p* = 0.034 and R= 0.58; *p* = 0.007, respectively) (Table 3, Figure 1 and Figure 2). ISI also was positively correlated with severity of IVH (*p* = 0.038). Pl-IPs did not show a relation with mean time <5 μV at day 2 (*p* = 0.558), but in the multivariable model, lower GA was associated with a longer mean time <5 μV (R = 0.60; *p* = 0.022).

## 4. Discussion

To our knowledge, this is the first study investigating the association between biomarkers of OS and quantitative aEEG/EEG measures in a cohort of extremely preterm infants. Our study demonstrated that patients with higher pl-IPs concentration at 24–48 h of life presented significantly longer ISIs and decreased SAT rates. The multivariable model showed that these correlations remained significant after adjusting for clinical risk factors. These results confirm our hypothesis of an association between lipid peroxidation products and neurophysiological maturity measured early after birth.

The aEEG/EEG features result from cortical network development that matures together with structural brain maturation during the later gestation [34]. SATs are the prevalent feature on the EEG of preterm infants. Their presence reflects the synaptogenesis of the thalamocortical axons induced by the migration from the subplate into the cortex [35,36]. Increased SAT rate has been associated with cerebellar and cortical gray matter maturation between 30 and 40 weeks of post-menstrual age (PMA) [37].

In preterm infants, during the cerebral development period from 23 to 32 weeks of PMA, pre-oligodendrocytes (pre-OLs) are the dominant cell type of the white matter [38]. It has been demonstrated that early brain activity might promote pre-OLs proliferation, differentiation, and myelin biosynthesis in the murine brain [39], while pharmacological blockade of pre-OLs differentiation prevented activity-regulated oligodendrogenesis and myelin development.

In addition, rodent models have shown that pre-OLs appear particularly prone to OS because of the lack of antioxidant mechanisms [18,19,20,40,41,42]. Activated microglia may release free radicals provoking the peroxidation of arachidonic acid of cell membranes via a non-cyclooxygenase mechanism, causing IPs production [43,44]. IPs are specific and valid markers of lipid peroxidation, and have been detected in biological fluids and in the autopsy brains of infants who suffered a brain injury [40,45]. In particular, increased levels of IPs have been demonstrated in human preterm autopsy brains during the early phases of WMI in association with a contemporary depletion of the pre-Ols. Furthermore, the IPs concentrations were similar to those found in the cerebral cortex after severe perinatal asphyxia in term infants. In addition, increased concentration of IPs has been encountered in the cerebrospinal fluid and on plasma of infants who developed WMI and severe IVH [32,46]. IPs are not only biomarkers of lipid peroxidation but also mediators of oxidant injury. IPs show bioactive properties as vasoconstriction of several vascular beds, including the brain [47], and it has been supposed that IPs may concur to neurovascular damage. Preclinical studies have shown that IPs may induce death of neuromicrovascular endothelial cell and pre-OLs by oncosis through thromboxane A_2_ (TxA_2_) synthesis [48,49].

We previously demonstrated that pl-IPs concentrations measured between 24 and 48 h after birth were significantly higher in preterm infants who later developed WMI evaluated on the MRI at TEA [23]. Findings of the present study have shown that pl-IPs levels are also associated with early cerebral function evaluated with aEEG/EEG, thus IPs are a reliable biomarker in structural brain development of the preterm brain.

Furthermore, ISI was positively associated with IVH staging (*p* = 0.038). Several studies indicated that depressed aEEG background activity could indicate the development of a large intracerebral hemorrhage or cystic periventricular leukomalacia in preterm newborns [10]. Moreover, in case of germinal matrix (GMH)-IVH, the amount of depression correlates with the degree of GMH-IVH [50,51].

We could not find a correlation between cb-IPs and early brain activity. Cb-IPs mirror oxygen radical exposure during fetal life and lipid peroxidation operated by placental metabolism. It has been described that IPs concentration changed following prenatal and perinatal causes, such as inflammatory reaction of the placenta, chorioamnionitis, fetal growth, perinatal depression, and birth weight [29,52]. In our model, GA was negatively associated with a longer time <5 μV on days 1 and 2. Consistently with previous research, in the present study, brain activity significantly modified with GA, with a higher SAT rate in infants with a higher GA [53].

This study has some limitations. First, the nature of the study was explorative, thus we did not calculate the power of the study. Second, the small sample size may have restricted the opportunity to identify a role of other factors influencing brain activity. Third, we investigated a specific form of brain injury that involves the prostaglandin metabolism. Other biomarkers of lipid peroxidation, such as neuroprostanes (IPs-like molecules derived from DHA), may be a more specific indicator of brain damage than F_2_-IPs, since DHA is the main polyunsaturated fatty acid in the brain [54,55].

## 5. Conclusions

Plasma IPs levels, measured between 24 and 48 h and adjusted for GA at birth, FiO_2_, and IVH are associated with early functional brain activity, expressed by SAT rate and ISI, showing higher levels in preterm infants with lower early electro-cerebral activity. Thus, pl-IPs may represent a useful biomarker of brain vulnerability in high-risk infants. These results, which need to be confirmed in larger study populations, could be used for early identification of high-risk infants and could have future implications for planning possible neuroprotective interventions in very preterm infants.

## Figures and Tables

**Table 1:** Clinical characteristics of the enrolled newborns. Mean (SD), number (percentage) or median (IQR).

	*n* = 39
Gestational age (weeks); mean (SD)	26.2 (1)
Birth weight (g); mean (SD)	859 (66)
Birth weight < 10th percentile; *n* (%)	2 (5)
Male; *n* (%)	17 (43)
Caesarean section; *n* (%)	18 (46)
Apgar score at 5 min; median (IQR)	8 (6–8)
Mechanical ventilation (days); median (IQR)	5 (2–16)
BPD; *n* (%)	5 (13)
PDA; *n* (%) Pharmacological treated Surgical closure	22 (56) 2 (5)
Sepsis; *n* (%)	7 (18)
NEC; *n* (%) Conservatively treated Surgery	1 (2) 3 (7)
IVH; *n* (%) 1–2 grade 3–4 grade	11 (28) 0 (0)
FiO_2_ between 24 and 48 h after birth; mean (SD)	24 (4)
WMI mild or moderate; *n* (%)	27 (69)
Cord blood isoprostanes (pg/mL); median (IQR)	59.3 (44.4–89.1)
Plasma isoprostanes between 24–48 h (pg/mL); median (IQR)	52.0 (33.2–82.2)

BPD: bronchopulmonary dysplasia; PDA: patent ductus arteriosus; Sepsis: culture proved sepsis; NEC: necrotizing enterocolitis; IVH: intraventricular hemorrhage; WMI: white matter injury, evaluated according to the scoring system by Woodward et al. [33].

**Table 2:** Univariate and multivariate linear regression analysis between cb-IPs and early brain activity at day 1.

Univariate Regression Analyses									
	ISI R = 0.26			SAT RATE R = 0.04			Pct Time Under 5 R = 0.10		
	B	95% CI	*p*	B	95% CI	*p*	B	95% CI	*p*
**cb-IPs**	−0.004	−0.013–0.005	0.344	0.001	−0.011–0.13	0.872	−0.025	−0.172–0.122	0.721
**Multivariate Regression Analyses**									
	**ISI** **R = 0.70**			**SAT RATE** **R = 0.45**			**Pct Time Under 5** **R = 0.67**		
	**B**	**95% CI**	***p***	**B**	**95% CI**	***p***	**B**	**95% CI**	***p***
**Gestational age**	1.1	0.37–1.8	0.007 *	−0.9	−3.4–1.6	0.419	−16.3	−28.7–−3.8	0.015 *
**BW (percentile)**	0.031	−2.82–0.114	0.407	0.40	−0.11–0.19	0.555	0.7	−0.3–1.9	0.152
**IVH (grade)**	−1.6	−5.7–2.4	0.380	−3.7	−11.4–3.9	0.289	−55.8	−114.5–2.8	0.059
**cb-IPs**	0.002	−0.013–0.016	0.815	0.012	−0.015–0.040	0.336	−0.101	−0.10–3.111	0.292

BW: birth weight; IVH: intraventricular hemorrhage; cb-IPs: cord blood isoprostanes; *: statistically significant *p* = < 0.05.

**Table 3:** Univariate and multivariate linear regression analysis: pl-IPs between 24 and 48 h after birth and early brain activity at day 2.

Univariate Regression Analyses									
	ISI			SAT RATE			Pct Time Under 5		
	R = 0.48			R = 0.56			R = 0.13		
	B	95% CI	*p*	B	95% CI	*p*	B	95% CI	*p*
**pl-IPs**	0.038	0.006–0.071	0.023 *	−0.039	−0.065–0.012	0.006 *	0.068	−0.170– 0.307	0.558
**Multivariate Regression Analyses**									
	**ISI**			**SAT RATE**			**Pct Time Under 5**		
	**R = 0.68**			**R = 0.58**			**R = 0.60**		
	**B**	**95% CI**	***p***	**B**	**95% CI**	***p***	**B**	**95% CI**	***p***
**Gestational age**	0.007	−0.377–0.391	0.971	0.1	−0.3–0.5	0.557	−7.5	−13.8–−1.2	0.022 *
**FiO_2_**	0.2	−0.2–0.7	0.105	0.2	−0.2–0.7	0.323	0.08	−2.0–1.9	0.931
**IVH (grade)**	1.6	0.1–3.2	0.038 *	−0.8	−2.8–1.0	0.332	4.3	−6.4–15.1	0.410
**pl-IPs**	0.030	0.003–0.058	0.034 *	−0.041	−0.069–0.013	0.007 *	0.139	−0.101–0.378	0.240

IVH: intraventricular hemorrhage; pl-IPs: plasma isoprostanes; *: statistically significant *p* = < 0.05.

## Data Availability

The datasets generated during and/or analyzed during the current study are not publicly available but are available from the corresponding author on reasonable request.
